# Management of Preventable Deaths due to Road Traffic Injuries in Prehospital Phase; a Qualitative Study

**Published:** 2019-06-24

**Authors:** Adel Eftekhari, Abbasali DehghaniTafti, Khadijeh Nasiriani, Majid Hajimaghsoudi, Hossein Fallahzadeh, Davoud Khorasani-Zavareh

**Affiliations:** 1 *Department of Health in Emergencies and Disasters, School of Public Health, Shahid Sadoughi University of Medical Sciences, Yazd, Iran.*; 2 *Department of Nursing, School of Nursing and Midwifery, Shahid Sadoughi University of Medical Sciences, Yazd, Iran.*; 3 *Trauma Research Center, Shahid Rahnemoon Hospital, Shahid Sadoughi University of Medical Sciences, Yazd, Iran. *; 4 *Department of Biostatistics and Epidemiology, School of Public Health, Shahid Sadoughi University of Medical Sciences, Yazd, Iran.*; 5 *Department of Health in Emergencies and Disasters, School of Public Health and Safety, Shahid Beheshti University of Medical Sciences, Tehran, Iran.*

**Keywords:** Death, emergency medical services, accidents, traffic

## Abstract

**Introduction:**

Prehospital care plays an important role in decreasing the number of deaths due to road traffic injuries (RTIs). This study aimed to identify the challenges of preventable deaths due to RTIs in the prehospital phase based on the attitudes of stakeholders.

**Methods:**

Conventional content analysis of qualitative study was used to analyze the data. The participants were 24 RTI prevention experts from fire-fighting organization, traffic police, the Red Crescent, Emergency Medical Services staff, emergency medicine specialists, and hospital emergency nurses who were selected by means of purposive sampling. Data were collected using unstructured interviews and analyzed by means of data coding, followed by extracting sub-categories, and main categories.

**Results:**

Six main categories were extracted as the major challenges of preventable deaths in RTIs in the prehospital phase including “poor management of the crash scene” with two subcategories of lack of rapid access to the patient and lack of scene safety, “lack of adequate rules and regulations” with two subcategories of lack of protocols and guidelines and lack of clear duties checklists, “poor management of time” with two subcategories of elongated response time at the crash scene and elongated time of victim transport, “low quality of training” with two subcategories of insufficient training of Emergency Medical Services (EMS) staff and inadequate public training, “poor communication and coordination” with two subcategories of poor communication of EMS staff and lack of uniform commandership at the crash scene, and “low quality of victim management” with two subcategories of low quality of clinical care and lack of accurate clinical assessment.

**Conclusion:**

The following measures are necessary to reduce preventable deaths due to RTIs in the prehospital phase: accurate clinical assessment of the victim on the scene, provision of high quality and accurate clinical care, enforcement of legal obligations and using protocols in the field of victim management, coordination of the involved organizations through identifying the duties and responsibilities of each organization, and full management of the crash scene by assigning a unique commander to each unit and creation of the highest level of safety on crash scene.

## Introduction

According to the World Health Organization (WHO), road traffic injuries (RTIs) account for about 1.3 million deaths worldwide annually and are the major cause of death among individuals aged 15-29 years (1, 2). The rate of mortality due to RTIs is constantly increasing in many countries, especially in low- and middle-income countries (LMICs) and 93% of all RTI victims live in such countries (3). Although fatal RTIs may occur at the crash scene, during patient transport, or in the hospital (4), according to some reports, most deaths occur in the prehospital phase in LMICs (5). Previous studies have indicated that 86% of trauma-related deaths occur in the prehospital phase (6); and about 39% of them are preventable (4). The presence of a systematic approach towards RTIs (7), and providing prehospital care aiming at higher quality emergency care to reduce victims’ mortality, play a pivotal role in effective management of these deaths (3). Moreover, selection of appropriate strategies and correct policy-making in these conditions may lead to decreased rates of death due to RTIs in different countries (8, 9). According to WHO, in most countries lack of sufficient information on preventable deaths in the prehospital phase is one of the major obstacles in suitable planning for reducing these deaths. 

Preventable deaths, as a concept, was first raised in 1970s and it was used as a method for measuring the quality of healthcare (10). Analysis of preventable death (PD) may aid in developing strategies for reducing mortality, improving quality of care, identifying new therapeutic strategies, applying advanced equipment, and enhancing training and technology (10, 11). This will also play a significant role in policy-making and care planning in the prehospital phase (4). Given the absence of a systematic approach in preventing the injuries and victim management after RTIs and obscurity of the weaknesses and strengths of prehospital emergency care in Iran, this study aimed to identify the current challenges that affect the preventable deaths management in the prehospital phase based on the stakeholders’ experiences.

## Methods

***Study design and setting***

This qualitative conventional content analysis study was carried out from March 2018 to November 2018. At first, the research goals and procedures and interview methods were elucidated to the participants who were assured of voluntary participation. They were assured of information confidentiality and anonymity of the participants and their official post or responsibility. The informed written consent form was signed by the participants regarding interview recording. They could withdraw from the study at any stage.

***Participants***

The study participants included experts and specialists at the national level including the Ministry of Health and Medical Education, and also at the provincial level of Yazd Province, Iran. The participants either had practical experience or theoretical knowledge on RTIs. They had at least five years of experience in RTI management in traffic police, the Red Iranian Crescent, fire-fighting organization, EMS, emergency medicine specialty, or hospital emergency nursing. Participants’ selection started and continued with purposive sampling method till data saturation was reached.

***Data Collection***

The first two interviews were carried out by means of unstructured in-depth interviews followed by 22 semi-structured interviews. The interviews were commenced by the open-response question covering: “What’s your opinion about preventable deaths in RTIs?” The follow-up questions were presented based on the feedback given by the participant and to clarify the concept under study. The items in the subsequent interviews were based on the concepts extracted from the data analysis. Some exemplar questions were: “What factors reduce the preventable deaths in RTIs? What strategies may be offered to reduce preventable mortalities in RTIs? In your opinion, what’s the role of various organizations in decreasing preventable deaths in RTIs?” The interview time was minimally 30 minutes and maximally 90 minutes with a mean time of 60 min. The interviews were first recoded digitally, and then transcribed verbatim on the same day using Word Office 2007.

***Data Analysis***

The digital voice files were listened to several times and the transcriptions were completed. Next, the interviews were reviewed several times and the meaning units were prepared. Subsequently, similar codes were merged and analysis units were reviewed several times and classified on the basis of the similarity and difference of conceptual and meaning. The codes with similarities were categorized together. Each time, the required modifications in the content and name of the category were done according to the item content. Finally, the researchers and experts agreed on the meaning of data, subcategories and main categories. The extracted subcategories were classified into larger categories until the main categories had emerged.

***Trusthwortiness***

In this study, the Guba & Lincoln criteria (12) were used to increase data accuracy and rigor. Therefore, the four criteria of Guba & Lincoln (1994), including credibility, transferability, dependability, and confirmability were taken into account by the researchers during the study process. To assure of the credibility, the researcher was in contact with the participants for a long time. Moreover, an attempt was made to select the participants with respect to their knowledge, experiences, work experience, work places, age, and gender. Increased dependability was achieved through step-by-step data collection and analysis and enjoying the reviews by experts and specialists. Furthermore, to enhance dependability, the reflectory commentary were repeated and the details were recorded. To ensure confirmability, the participants’ opinions were checked to confirm the accuracy of codes by means of member check. If there was any mismatch between the codes and the participants’ opinions, the required corrections were made. Furthermore, the dyadic control was done by two faculty members and experts in qualitative studies (D. Kh; Kh, N.) and agreement was achieved on the distilled codes and categories. To make sure of transferability, the details of the study were mentioned. Finally, to establish confirmability, investigator triangulation was also used for checking the codes sub-categories and categories.

***Ethical Considerations***

This study was part of a PhD thesis in Health in Emergencies and Disasters that was approved by Ethics Committee of at Shahid Sadoughi University of Medical Sciences with ethics code no.: IR.SSU.SPH.REC.1397.038 dated 18.6.2018. 

## Results

A total of 24 experts aged 31-55 years were interviewed (62.5% male). Baseline characteristics of participants are given in Table 1. The official posts of the participants included: traffic police, Red Crescent staff, fireman, EMS technician/staff, EMS specialist, emergency nurse, specialist in Health in Disasters, and coroner or forensic medicine specialist. 

Six main categories were extracted as the major challenges of preventable deaths in RTIs in the prehospital phase including “poor management of the crash scene” with two subcategories of lack of rapid access to patient and lack of scene safety, “lack of adequate rules and regulations” with two subcategories of lack of protocols and guidelines and lack of clear duties checklists, “poor management of time” with two subcategories of elongated response time at the crash scene and elongated time of victim transport, “low quality of training” with two subcategories of insufficient training of EMS staff and inadequate public training, “poor communication and coordination” with two subcategories of poor communication of EMS staff and lack of uniform commandership at the crash scene, and “low quality of victim management” with two subcategories of low quality of clinical care and lack of accurate clinical assessment. Table 2 presents the main categories and subcategories of challenges influencing preventable deaths in RTIs in the prehospital phase on the basis of participants’ opinions in Iran.

**1. Poor management of the crash scene**

Lack of Rapid Access to Patient

Before the rescue team arrives at the scene, the victim should be made ready for intervention and the rescuers or first aiders only perform an initial assessment of the victim and provide the required care instead of wasting their valuable time in anticipating for other organizations to place the victim in the appropriate situation. Therefore, all interventions for victim preparation should be done before the rescue team arrives. In the participants’ viewpoints, the prompt attendance of Red Crescent staff at the crash scene, having standard equipment and facilities, and sufficient skill and experience in disentangling the victims are among the factors that play an important role in quick preparation of the victims for prehospital emergency interventions.

**P21:** *“…sometimes a car overturns in a road accident and is demolished, but when I arrive at the crash scene as an EMS technician, I can see that the Red Crescent has not been able to untrap the victim out of the car wreckage. They really need training and expertise…”*

**Lack of scene safety**

The participants believed that lack of scene safety can serve as an influential challenge affecting the management of preventable deaths in RTIs. Though establishing the scene safety has been rendered as the duty of the police, in some cases the quarrel between people and the rescue team, people and the police, or the rescue team and the police has impaired scene safety leading to tension at the crash scene. Lack of prompt presence of the police at the crash scene and occasionally lack of familiarity of the police with their duties or absence of shared understanding between the police and EMS staff have caused tension at the crash scene, negatively affecting EMS performance and thus hindering the immediate care-giving at the crash scene. Prehospital emergency care-giving environment is stressful itself and affects EMS performance. In addition, people get anxious on the crash scene, they observe the victims and give some advice to EMS staff about care-giving; however, this impairs the EMS staff’s concentration adding to the stress governing them on the crash scene. That is why the police force should provide suitable conditions for the rescuer organization so they can give the necessary primary care as fast as possible and transport the victim. Since the police are the prime managers of the scene safety, they must perceive the effect of the critical condition on the EMS staff and control the bystanders at the crash scene without any quarrel or involvement. On the other hand, the medical and emergency equipment or materials in the ambulances are sometimes stolen at the crash scene by the passersby indicating lack of safety at the crash scene.

**P12:** *“…when we arrive at the crash scene, we face some angry people; I just think they’re right and their anger is justified; yet, it’s the duty of the police to manage the people and take them away from the scene of the injury. They should provide a safe scene for me to work properly and avoid pile-ups by stopping the traffic at least 200 meters away from the crash scene. However, the police sometimes do not engage themselves and I have to work under so much stress…”*

**Table 1:** Baseline characteristics of participants

**Variables**	**Number (%)**
**Age (year)**	
< 40	4 (17)
40-50	13 (54)
> 50	7 (29)
**Gender**	
Male	15 (63)
Female	9 (38)
**Education level**	
Associate diploma	2 (9)
BS and MSc	7 (29)
PhD/specialist	15 (62)
**Work experience**	
< 15	4 (17)
15	3 (13)
>15	17 (71)

Bs: Bachelor of science; MSc: Master of Science; PhD: Doctor of philosophy.

**Table 2:** Challenges affecting the management of preventable deaths due to road traffic injuries in the pre-hospital phase based on the participants’ opinions in Iran

**Categories**	**Subcategories**	**Code examples**
Low quality of victim management	Low quality of clinical care	-Wrong intubation -Insufficient fluid therapy of patients -Not using cervical collar for the victims
	Lack of accurate clinical assessment	-Inadequate report of medical history of patients to the hospital by technicians -Incomplete clinical examination of the patients by technicians -Unsuitable diagnosis of clinical status due to negligence in victim survey
Poor management of the crash scene	Lack of rapid access to patient	-Lack of prompt presence of staff of the Red Crescent at the scene -Lack of sufficient skills in Red Crescent staff for rapid releasing of the victim -Lack of adequate equipment for the Red Crescent staff in releasing the victim
	Lack of scene safety	-Necessity of supervision on ambulance equipment to prevent robbing of equipment by bystanders at the scene -Lack of proper cooperation of the police to prevent quarrels at the crash scene -Physical fight between angry people and EMS technician at the crash scene
Poor communication and coordination	Poor communication of EMS staff	-Deficiency of communication infrastructures -Necessity of teamwork by prehospital rescue team
	Lack of uniform commandership at the crash scene	-Indecisiveness of the rescue team at the crash scene -Irrelevant interference and assertions by different bystanders at the crash scene -Dominance of the police over the crash scene most of the time
Lack of adequate rules and regulations	Lack of protocols and guidelines	-Necessity of reviewing the educational curriculum of Medical Emergencies major -Necessity of assessing educational needs of prehospital rescue team -The need for use of experienced and tactful staff in prehospital emergency care -Necessity of continuous and up-to-date education of the staff providing prehospital emergency care
	Lack of clear checklists for duties	-Necessity of providing public education for dealing with road traffic injuries -Lack of familiarity of the public with first aid principles -Secondary damage to the victims by wrong interference of irrelevant individuals at the crash scene
Poor time management	Elongated response time at the crash scene	-Time-consuming path to the crash scene due to unfamiliarity of the staff with uncrowded routes -Lack of collaboration of people in opening the way for the ambulance -Lack of management of the police to avoid pile-ups at the crash scene
	Elongated time of victim transport	-Wandering of ambulances at the crash scene due to lack of exit path for ambulances -Not opening the road for ambulances by people from the crash scene to the hospital
Low quality of training	Insufficient training of EMS Staff	-Necessity of revising educational curriculum for EMS major -Necessity of assessing needs of prehospital EMS staff -Necessity of using skilled and tactful clinical staff in prehospital emergency care -Necessity of up-to-date training of prehospital rescue team
	Inadequate public training	-Necessity of providing public education on proper approach to road accident victims -Unfamiliarity of the public with first aid principles -Secondary damage to the victims due to the wrong interference of irrelevant individuals

EMS: Emergency medical service.

**2. Lack of adequate rules and regulations**

**Lack of protocols and guidelines**

Lack of comprehensive protocols and guidelines in all areas related to prehospital EMS (managerial and therapeutic) of RTIs has been mentioned by the participants as another major challenge affecting preventable mortalities of RTIs. There are no clear-cut guidelines for many cases like management of first aid bases, care-giving to traumatized patients in the hospital and prehospital phases, as well as guidelines of common act and extra-organizational communication. The participants have rendered as necessary the obligation for revising guidelines and protocols and updating them and also development of offline protocols for various situations to facilitate the decision-making of the responding staff. The offline protocols are required for performing any therapeutic interventions or managerial control in the prehospital phase. These offline protocols are needed for uniform and standard scientific treatment and care-giving and promotion of high quality care in EMS missions.

**P17:** *“…we need protocols, especially for ATLS and PTLS, which we have no protocols for; even hospitals don’t have these protocols. The staff members need guidelines showing them what to do, for example, if communication was cut off, I mean the offline protocol…”*

**Lack of clear duties checklists**

The participants believed that a detailed checklist of duties in the prehospital phase of RTIs was necessary for each organization to indicate the role of each organization in case of injuries. This issue has been largely ignored by the authorities. Indeed, obscurity of roles and duties of organizations (the Red Crescent, the police, and the fire-fighting organization) can, in its own account, serve as an influential challenge affecting preventable deaths. When a number of organizations agree to achieve a common goal, an important thing to do is proper management of those systems and determining their duties. Given the sensitivity of the concept of time in the crash scene, the detailed identification of duties will be very influential, which if left unnoticed, it will result in parallel work.

**P9:** *“…when a force comes to the crash scene, they should be aware of their duties and oriented with them. Many times we’ve entered the crash scene and observed that the police don’t know what to do…”*

**3. Poor time management**

**Elongated response time at the crash scene**

The participants stated that the time of arrival of ambulance at the crash scene of RTIs and initiation of therapeutic interventions for the victim are highly important in avoiding preventable RTIs deaths. However, they believed that victims do not receive the required treatment even in the case of preparation for giving high quality care due to reasons such as shortage of civil EMS bases, the route traffic jam, lack of special route for rescue cars, and underdeveloped air ambulances. The prehospital rescue staff believed that if the green ring way, i.e. the shortest route for reaching the crash scene, is not announced by the police, this makes the EMS staff to select the route themselves occasionally leading to selection of a wrong route with high traffic jam. The participants stated that the pile-ups near the crash scene where drivers stop their cars to film the crash scene or victims inquisitively elongates the access time for the prehospital EMS staff. Opening the route to the crash scene should be done by the police; however, the EMS staffs face a crowded crash scene with much pile-up due to disorientation and negligence of the police.

**P17:** *“…sometimes, there is much traffic jam in the city and drivers do not pay attention to the ambulance siren, so, we have to take short-cuts to reach the crash scene sooner. Yet, very often we face some problems in those short-cuts. Suppose you are facing a crash scene in the crowded Tehran-Qom highway. There is traffic jam 4 km before and 4 km after the crash scene; how can I reach the crash scene to access the victim…?”*

**Elongated time of victim transport**

Another subcategory of poor time management is elongated time of victim transport. Delay in announcing the injury to the hospital, delay in arrival at the hospital, and delay in delivery of the victim to the hospital are challenges that affect the rate of preventable deaths in RTIs. The EMS Staff should deliver the victim to the hospital emergency physician along with the required medical history. However, when the prehospital emergency staff members arrive at the hospital, they should wait a long time to deliver the victim to the emergency physician, although the critically ill victim is in the ambulance or on the stretcher in the emergency department. This is due to the fact that they give priority to other victims in the triage and ignore the RTI victims.

**P13:** *“…when we take the victims to the hospital and want to deliver them, we wait a long time and no one is responsible…”*

Each EMS system has a physician called the guide physician who is legally responsible for the clinical aspects of patient care. The therapeutic interventions performed in the prehospital emergency care are done by the permission of this guide physician. When there is a critically ill victim in the crash scene and the EMS staff has to receive the required instructions from the guide physician via the wireless, a 1-second delay in responding may lead to victim’s death. From the participants’ view, prompt and effective responding of the on-call physician in appropriate provision of clinical care is directly related to the victim and any delay in the on-call physician’s response interrupts care-giving to the victim.

**P13:** *“…it has happened quite often that we called the on-call physician, but nobody responded; in many places, our wireless or cell phone had no signal…”*

**4. Low quality of training**

**Insufficient training of EMS staff**

The participants asserted that training is one of the important factors in increasing skillfulness and tactfulness of EMS staff; however, there are some significant challenges in this regard. Challenges, such as lack of attention to comprehensive training during the retraining courses, lack of accurate assessment of requirements in line with the prioritized cases needed by EMS staff at the crash scene, and absence of continuous educational evaluations for the staff has caused the EMS staff to not possess the required skill and ability to give proper clinical care on the crash scene.

**P6:** *“…some of the retraining courses held in the system are not useful for us; we just join such classes for their educational points and privileges…”*

The employment of the staff in prehospital emergency care unit should be done on the basis of certain educational frameworks and rules. Recently, the lack of any educational monitoring system for the employment of EMS staff has led to the employment of staff with various academic degrees, such as phlebotomist, nurse, associate diploma/BS of EMS, and 6-month trained technicians. This has led to a gap between knowledge and skills among the staff; therefore, each group needs to receive suitable educational services.

**P8:** *“…6-month trained staff who have no sufficient knowledge and skill in care-giving to victims are employed. This causes secondary damage to victims in some occasions…”*

**Insufficient public training**

The participants believed that sometimes lack of sufficient knowledge among people, performing the wrong interventions before the EMS arrives at the crash scene and improper measures during care-giving imposes irreversible damages on the victim indicating the importance of training for the public. Sometimes, the intervention done for the victim by the first bystanders in the crash scene enhances the performance of EMS staff; nevertheless, many times such interventions lead to more severe damage to the victim that may be irreversible. From the prehospital emergency care staff’s viewpoint, public training in areas like medical emergencies, making contact with EMS facilities, and anything required for primary treatment of the victims ought to receive special attention.

**P7:** *“…we had a victim on the crash scene with a great amount of blood in the throat. People at the scene did not know that a simple change of position may remove the secretions, clear his airway and save him; unfortunately, the victim died…”*

**5. Poor communication and coordination**

**Poor communication of EMS staff**

From the participants’ perspective, proper communication between the staff during performing duties is always important in achieving the intended result. Factors, such as poor communication between EMS Staff, between EMS staff and Red Crescent staff, and between police and fire station staff serves as an influential challenge affecting the preventable deaths in RTIs. Absence of a common jargon among the EMS Staff, lack of agreement on priorities and concepts, weak infrastructures and communication equipment, and lack of communication paths were mentioned as major obstacles in the way of rapid and efficient communication.

**P10:** *“…the police can communicate with EMS system, but I can’t access them since this communication is unilateral. In many occasions, there has been the need for communication yet, I didn’t have any access to police…”*

**Lack of uniform commandership at the crash scene**

Absence of uniform commandership at the crash scene was mentioned as one of the effective challenges in care-giving to victims from the participants’ perspective. Lack of integral commandership at the crash scene makes the forces function diversely as separate islands without any coordination among them leading ultimately to low quality care. Different people at the crash scene order the prehospital EMS staff with their dos and don’ts or musts and mustn’ts; while there are sometimes obvious contradictory orders. Sometimes, the involved organizations do not show much compliance towards each other predisposing to indecisiveness of the forces.

**P14:** *“…everybody at the crash scene states their own opinion with nobody there to obey them. They all want to say that their opinion is correct…”*

**6. Low quality of victim management**

**Low quality of clinical care**

The prehospital emergency care staff believed that the type and priority of the provided care, promotion of quality care, and its warranty are of utmost importance; therefore, they asserted that the mere provision of care would not suffice; this care ought to be given with proper quality and via observation of professional knowledge too. They also stated that the provided care sometimes does not possess the required quality in the prehospital phase and negligence in care provision at the crash scene or during victim transport is an important challenge itself in managing preventable deaths in prehospital emergency care.

**P8:** *“….it has happened several times that when I received the victim from the prehospital care team, I saw that the IV-line placed for the patient was, in fact, subcutaneous or I observed several times that they had tried to perform intratracheal intubation for the patient, but they did intraesophageal intubation….”*

**Lack of accurate clinical assessment**

From the participants’ viewpoints, inappropriate assessment of the clinical condition of victims on the crash scene or during transport and absence of subsequent accurate evaluations at the time of delivery of the victim to the hospital or inadequate victim examination by the physician play a significant role in increasing preventable deaths in RTIs. Some prehospital care staff members believe that a rapid and simple cephalocaudal examination of the patient should be done leaving the complete examinations for the hospital emergency and physicians. Rapid transport of the patient to hospital is important; however, the sufficiency of prehospital emergency interventions on the victim is also important. The patient’s condition ought to be assessed thoroughly; otherwise, any negligence may predispose the patient to death.

**P7:** *“….when the victim is brought in by the technician and I ask him/her ….about their clinical condition, I see that he/she is not aware of it; for example, he/she does not know their score on Glasgow coma scale or where in the body the patient has trauma. They can’t answer my questions; then I understand that they have not checked the victim sufficiently…”*

**Discussion:**

This study investigated the managerial challenges of preventable deaths in RTIs in the prehospital phase. It was indicated that the prehospital phase of RTIs suffers from deficiencies like low quality of victim management, poor management of the crash scene, shortage of communication and coordination, lack of required rules and regulations, poor management of time, and low quality of training. Paying attention to these challenges would lead to provision of high quality care to victims in the prehospital phase.

Conventional content analysis is a suitable method for obtaining valid and reliable results using transcribed text data. It creates knowledge, new ideas, and facts and serves as a practical guide of performance (13). It may prove useful in deep recognition of challenges affecting preventable deaths in RTIs in the prehospital phase.

One challenge found in this study that affects the management of preventable deaths in RTIs was low quality of clinical care and lack of accurate clinical assessment of the victims in the prehospital phase. Suitable primary care for traumatized victims on the crash scene and during transport improve the poor prognosis of the victims by itself, although it has been proved to be effective in decreasing the severity of damages to the victims (9, 14-17). Interventions like airway management, intubation, and chest tube placement are considered advanced care on the crash scene (18). In a study that investigated the quality of prehospital care for traumatized patients referred to emergency of a hospital in Tehran, wound dressing and hemostasis were performed correctly in 80% of cases. Splint was also applied correctly in 50% of cases while interventions, such as cervical collar and spinal bed were not performed in 80% of cases. Furthermore, although the EMS staff had placed an IV-line in 91.2% of cases, no serum was infused to the victims in 80% of cases (19). Prehospital emergency care should be not only rapid, but also accurate and precise (20). Indeed, appropriate interventions on the crash scene are one of the cornerstones of prehospital emergency care. High quality care-giving is the main factor in saving humans’ lives, which is considered the best outcome.

 Another challenge postulated by the participants was the lack of accurate clinical assessment of the victims in the prehospital phase of RTIs. Joshipura et al. enumerated 12 priorities as care priorities in injuries, including team formation, airway investigation, respiration, blood circulation and shock, diagnosis and monitoring, head and neck injuries, chest and abdomen, upper and lower extremities, spinal cord, burns and injuries, pain control, rehabilitation, and infection control each of which ought to be prioritized depending on the type of RTI (21). Providing optimal care for victims and determining life-threatening conditions demand accurate evaluation. Precise clinical evaluation is one of the most important ways of obtaining vital information on the victims, which is unfortunately ignored most of the time or performed hastily. In fact, prehospital emergency care deals with making decisions about treatment and transport. The victim assessment data are the guiding factor for EMS staff in their decision-making at the crash scene. The staff should perform a primary assessment of the vital signs of the victim or any life-threatening factors after they are assured of the safety of their location and safety of the victim. This assessment ought to be highly accurate so that it can completely identify the status of the victim when the diagnostic data are conveyed to the on call physician.

Poor management of the crash scene was another influential challenge affecting the preventable deaths in this study. Poor management is one of the current challenges of RTIs in Iran. In this study, delayed access to victim was one of the challenges that affected deaths related to RTIs. This factor is influenced by factors, such as crowded pile-up and closure of access route to the crash scene. Passerbys’ intervention at the crash scene either facilitates or debilitates preventive care in RTIs (22). The public presence at the crash scene due to personal curiosity, enthusiasm for humanistic help, excitement, and haste result in a crowded pile-up and poor communication (23). Providing primary emergency care on the crash scene and during victim’s transport to hospital help to improve victim’s prognosis by itself. WHO recommends scene safety for road accident victims (9) as the primary intervention (24). Various organizations collaborate at the crash scene to achieve the common goal of saving the victim’s life. The Red Crescent staff members are among the first aiders who should be present at the crash scene as securers of scene safety before the EMS staffs arrive. They should prepare the victim for receiving care. Considering the importance of time and the urgency of performing therapeutic interventions for victims, the crash scene should be set in a way that provides immediate victim access for prehospital EMS technicians. The situation should be adjusted by the Red Crescent staff that arrives at the crash scene before the EMS staff, so that the EMS staff can have access to victims once they arrive there. Moreover, some studies have demonstrated that illogical and irrational public pile-up at the crash scene elongates the course of care-giving to victims and reduces safety (9, 25, 26). The crowd wastes the critical time needed for prompt care-giving and may even induce secondary damages and even cause new injuries (27). Lack of proper communication and coordination was another variable affecting preventable deaths presented by the present study. Suitable communication and coordination can increase readiness for prehospital care provision (17, 28). Lack of coordination in the healthcare system has led to improper allocation of budget to healthcare and deprivation of patients form adequate care (15). A balance should exist between human resources and communication equipment. The victim shall receive optimal care when there is a close contact among all emergency care providers. The public crowd at the crash scene doubles the stress, which leads to reduced collaboration among the EMS staff and hinders sharing of experiences for performing therapeutic interventions on victims and controlling the crash scene. Another factor required for coordination between the EMS staff and physician is the presence of appropriate equipment. The use of a widespread communication system, creation of an effective direct wireless system, and working with a uniform frequency for all administrative and supervising respondents at the crash scene are mandatory. Lack of a uniform commandership system is another factor related to the shortage of communication and coordination needed for proper management of preventable deaths in RTIs. Depending on type of injuries, various organizations attend the crash scene; hence, there ought to be a uniform comprehensive system of commandership to manage the scene with due power. The accident commander should act in a way that enables him/her to actively manage all of the traffic trends during the accident till the situation returns to normal. The police have been introduced as the coordinating element in managing RTIs as well as factors that influence crash scene management (29, 30). The accident commander may vary depending on the type of accident. Nevertheless, the important point here is that, due to the presence of different organizations, the commander can be selected on the basis of injury type through developing the required processes and guidelines. Another challenge identified in this study was the lack of rules and regulations, which brings about indecisiveness in care-giving by EMS staff and sometimes performance of incomplete or repetitious interventions due to absence of checklists for duties. Additionally, another challenge in prehospital emergency care was the presence of diverse guidelines and protocols without considering clinical evidence. The presence of developed protocols and guidelines are deemed mandatory for promoting communication, better clinical care, and the subsequent decrease in RTI mortality (31). Lack of evidence-based protocols and guidelines would lead to lack of standard clinical care provision and disintegration of communication and coordination process in management of the crash scene, resulting in increased preventable deaths in RTIs (26). On the other hand, the present protocols mostly emphasize the monitoring of victims with few of them dealing with clinical care provision (32). Today, one of disasters of prehospital emergency care is the lack of checklists for duties of each organization involved in RTIs. When a number of rescue organizations join together to achieve a common goal, proper organization of these systems and determining the details of their duties are obligatory. Various systems such as the police, emergency service, the Red Crescent, fire fighting organization, etc. collaborate with each other depending on the type of injury. In so doing, they require a checklist of duties for each organization to both prevent interference and enable each to recognize its own duties on the basis of the present conditions. 

The present study rendered poor time management as an important challenge in prompt clinical care provision to RTI victims. In this regard, elongated response time and transport time are considered as important challenges of time management. The kind of response by bystanders at the crash scene influences the EMS response time. The findings of previous studies suggest that reduced time at the crash scene improves the outcome of RTI victims (26, 33). Exploration of the status of traumatized victims in the prehospital emergency care reveals that the response time in RTIs has decreased in Iran and the emergency care approaches the global standards in most Iranian cities except Tehran, the capital (34, 35). Despite the fact that time at the crash scene for stabilizing the traumatized victims would improve their prognosis (36-39), the American College has recommended that the stay time at the crash scene should be reduced as much as possible (33). Earlier coordination between the prehospital and hospital emergency care units is one of the influential factors that decreases the victim transport time and enhances the treatment team’s preparation for proper care-giving (40). One of the most influential factors affecting the standard response time is a crowded access route since the current traffic jam attenuates the efficacy of ambulances and their fast performance. This causes the response time to exceed the standard limits. Heavy traffic jam is reported as one of the reasons of elongated response time in other studies (41, 42). The use of motorcycle ambulance and air ambulance is recommended for quick transportation of victims from the crash scene to the hospital emergency care indicating the importance of saving human lives. Another factor leading to an elongated EMS response time is the long distance. If the ambulance driver is not familiar with the short-cut routes, they will certainly arrive late at the crash scene. Some strategies such as the technician’s familiarity with short-cut routes, the use of satellite path-finder systems, radar, GPS, and high frequency waves for finding the best route in emergency cases can help in this regard (42). Of course, it should be kept in mind that finding the shortest route is not always possible and sometimes a low traffic but longer route with higher speed can be more helpful. 

Another challenge extracted in this study was the lack of effective training of EMS staff and the public as the first responders at the crash scene for reducing preventable deaths in RTIs. It is highly important to plan suitable educational programs on preventing RTIs and quick response to them. Assessment of needs should be prioritized first, followed by dynamic training and updating staffs’ knowledge in a concentrated framework of management and organization. In a study conducted on assessing educational needs of EMS technicians, most of them pointed to knowledgeablility followed by pharmaceutical information and drug complications. Regarding skills, they pointed to skillful intratracheal intubation, and with respect to attitudes, they pointed to participation in in-service continuous educational courses for promoting their practical skills (43). Topics on prehospital emergency care such as crash scene assessment, identification of work groups in emergency room (ER), identifying details of patient examination, and triage ought to be covered in the syllabus of would-be rescuers such as students of nursing or EMS (44). Use of simulation in the virtual form or using a computer or online method in a recreated environment will be helpful in improving the victim’s management on the crash scene, increasing the ability of scene management, and enhancing decision-making. Moreover, the use of “standard patient” will be beneficial in training the staff on how to treat traumatized patients with internal problems, or symptoms of special disorders. Even role playing can help in training and creating the feeling required for the role of learners to approach patients under different conditions. Holding practical classes during the education years may positively influence the volunteers of working in emergency wards. These classes should be held on the victims’ bedside to be more efficient (45). To train the EMS staff, emphasis should be on experienced instructors, revision of educational classes, and the use of practical methods of training wile also considering the results of assessment of needs and the quality of interventions. Furthermore, EMS bases should be equipped with educational technology such as moulages, TV and video players, along with teaching materials like pamphlets, books, booklets, etc. The important topics to be covered in prehospital emergency care syllabus include crash scene assessment, identification of work groups in emergency care, identification of details of patient survey, and triage (46). Provision of the required training in clinical skills will promote the work quality of EMS staff and other organizations involved. Avoiding first aid provision by the public and lack of training in this regard was found to be another important challenge in this study. Training should be based on the specific needs of each community and the available resources. Training should use simple local language and focus on transferring vital skills of crash scene management, hemostasis, primary Cardiopulmonary resuscitation (CPR), handling and transporting the victim with open fractures, and triage. The acquisition of these skills by these learners,is highly efficacious immediately after injuries in the absence of healthcare experts. A study on prehospital emergency care obstacles conducted in LMICs (2018) reported lack of training as one of the known obstacles in the way of better care provision in the prehospital phase (15). Moreover, public education regarding the crash scene and the acquisition of first aid skills for improving victim outcomes in the prehospital phase are mentioned as effective factors (35, 44). The role of bystanders as primary helpers at the crash scene has been emphasized (17). Some countries like India plan and implement special courses for training the public for medical intervention in emergency scenes (23). Indeed, development of public education in first aid at the crash scene is mandatory. Of course, direct provision of first aid will not work without preparing the cultural background and infrastructures. Then public would wish to learn and the healthcare system would inform the public about the importance of saving lives.

## Limitation

The limitation of this study was that it was difficult to coordinate interviews with experts. Time limitations were other limitations of the study. Persistent attempts by the researcher resolved this issue to some degree.

## Conclusion:

The participants' experiences about challenges that influence management of preventable deaths in RTIs showed that, in order to improve management of possible deaths resulting from it, consideration of issues such as accurate clinical assessment of the victim on the crash scene without any negligence, provision of high quality care, the presence of required legal obligations and protocols for treatment and management, coordination of the involved organizations through identifying detailed duties for each, and full management of the crash scene via determining a uniform and unique commander and creating the highest level of scene safety are essential. The authorities should take into account the scientific knowledge and practical skills of EMS staff, and public first aid training regarding the crash scene.
